# Modification of the susceptibility gene TaPsIPK1 - a win-win for wheat disease resistance and yield

**DOI:** 10.1007/s44154-022-00060-3

**Published:** 2022-09-20

**Authors:** Alberto Macho, Pengcheng Wang, Jian-Kang Zhu

**Affiliations:** 1 Shanghai Center for Plant Stress Biology, CAS Center for Excellence in Molecular Plant Sciences, Chinese Academy of Sciences, Shanghai, 200032 China; 2 Institute of Advanced Biotechnology and School of Life Sciences, Southern University of Science and Technology, Shenzhen, 518055 China; 3 Center for Advanced Bioindustry Technologies, Chinese Academy of Agricultural Sciences, Beijing, 100081 China

**Keywords:** Wheat, Wheat stripe rust, *TaPsIPK1*, Resistance genes

## Abstract

Wheat is one of the most important cereal crops, and it is essential for worldwide food security. However, wheat production is threatened by various diseases, including wheat stripe rust caused by the fungus *Puccinia striiformis* f. sp. *tritici* (*Pst*). The development of plant resistance against disease is usually challenged by potential reduction in crop yield due to the enhancement of plant immunity. In a recent article, Wang et al. found that *TaPsIPK1* is a susceptibility gene targeted by rust effectors. Editing of *TaPsIPK1* increases resistance to stripe rust without any developmental effects or yield penalty, providing an exceptional resource for developing disease resistance in wheat.

This brief article highlights the recent results of Wang et al. (2022), who found that knockout of a susceptibility gene increases resistance to stripe rust of wheat without yield penalty. Wheat, one of the most important and widely cultivated cereal crops, provides approximately 20% of the dietary calories and protein for humans worldwide (Shiferaw et al., 2013). Because the global human population is estimated to increase to nearly nine billion in 2 to 3 decades, ensuring food security will require an annual increase of 2% in wheat production (Rahmatov, 2013). The limited availability of suitable land means that increases in wheat production will require increases in yield/ha. Such increases will be difficult to achieve given the substantial challenges to wheat production represented by various diseases, including wheat stripe rust.

Wheat stripe rust, also known as yellow rust, is caused by the fungus *Puccinia striiformis* f. sp. *tritici* (*Pst*) and severely threatens wheat yield, grain quality, and forage value (McIntosh et al., 1995; Carmona et al., 2019; Liu et al., 2017). Most wheat producing regions in nearly 60 countries are vulnerable to this disease, which can cause 5–25% yield losses (Chen 2005; Wellings 2011). In Asia, up to 46% yield losses are due to stripe rust epidemics (Singh et al., 2004). Stripe rust caused more than 40% losses of China’s total wheat production in the 1950s (Chen et al., 2014), and the four destructive epidemics that occurred between 1950 and 2002 caused yield loss up to 12.3 million tons (Wan et al., 2007). From 1958 to 2016, stripe rust caused yield losses of over 31.7 million tons in the USA (Chen and Kang, 2017). Therefore, wheat stripe rust is a significant constraint for wheat production and a threat to global food security.

The most effective way to control rust diseases is by planting and breeding durably resistant wheat cultivars. Conventional breeding for disease resistant crops takes advantage of disease resistance genes (*R* genes), which confer strong resistance when the plant detects effector proteins secreted by the pathogen. Substantial research has been carried out to identify and clone stripe rust resistance genes, commonly referred to as wheat yellow rust resistance genes (*Yr*). To date, over 80 *Yr* genes have been identified (Wu et al., 2018), among which *Yr1*, *Yr9*, and *Yr26* have made great contributions to the prevention and control of wheat stripe rust (Wan et al., 2007). However, *R* gene-dependent disease resistance frequently loses effectiveness when pathogen mutations allow the pathogen to evade detection by host plant immune receptors. As a result, many of the identified *Yr* genes have become less effective and are no longer used in China or elsewhere. Unlike *R* genes, host susceptibility genes (*S* genes) are exploited by the pathogen to promote the infection, and therefore, disruption of host susceptibility by inactivating *S* genes can result in disease resistance. Hence, mutating *S* genes has become an emerging and powerful approach for breeding durable resistance (Pavan et al., 2010). However, relative to the extensive research on wheat *R* genes, research on wheat *S* genes has been quite limited.

As a typical obligate biotrophic fungus, *Pst* relies on living wheat plants to survive; it absorbs nutrients from host cells through haustoria, and releases effectors into host cells, which promote disease development by suppressing the plant immune system and manipulating host *S* genes. Rather than identifying *R* genes, a group of researchers from Northwest A&F University (Xianyang, Shaanxi, China) has been investigating the interaction between wheat and *Pst* with the aim of exploiting key *S* genes for wheat breeding. This group recently identified a novel wheat *S* gene, *TaPsIPK1*, and reported that the loss of function of *TaPsIPK1* confers broad resistance of wheat to stripe rust (Wang et al., 2022). *TaPsIPK1* is a cytoplasmic receptor-like kinase gene, which was first identified among the differentially expressed genes during the compatible interaction between wheat and *Pst*; transient silencing of this kinase gene decreased wheat susceptibility to *Pst*. *TaPsIPK1* has three homeologs in hexaploid bread wheat (*TaPsIPK1*-6A, *TaPsIPK1*-6B, and *TaPsIPK1*-6D), all of which are induced upon infection by virulent *Pst*. Their simultaneous silencing by RNA interference resulted in increased resistance to *Pst*. Importantly, Wang and colleagues used CRISPR/Cas9 gene editing to generate a stable knockout line in wheat that lacks all three *TaPsIPK1* genes. These triple *TaPsIPK1*KO mutations conferred resistance to all of the main races of *Pst* that cause epidemics in China, as well as to the leaf rust fungus *P. triticina.* During the stripe rust pandemics of 2020 and 2021 in China, *TaPsIPK1*KO plants maintained effective resistance to *Pst*, producing relatively higher yields in the field. In contrast to the race-specific resistance mediated by dominant *R* genes, resistance mediated by the disruption of *TaPsIPK1* is not race-specific, and thus provides excellent wheat germplasm for breeding durable resistance in wheat. The study of Wang et al. (2022) is the first report on the identification of a novel susceptibility gene that can be modified for conferring broad spectrum resistance against rust diseases that threaten wheat production and global food security.

Because *S* genes usually have important physiological roles, mutation of *S* genes often leads to unexpected consequences, such as yield penalties that are commonly observed in wheat and other crops. It is interesting that inactivation of *TaPsIPK1* enhances wheat resistance but does not cause any apparent developmental defects. Wang and colleagues characterized the immune responses mediated by deletion of this *S* gene and showed the critical role of TaPsIPK1 in negatively regulating PTI (PAMP-triggered immunity) as well as ETI (effector-triggered immunity). Inactivation of *TaPsIPK1* increased constitutive resistance, showing higher levels of *TaPR1* expression and SA accumulation compared to those in wild type (WT) plants, yet these changes are subtle enough not to trigger cell death. Moreover, these plants showed faster and stronger defense induction than WT plants upon *Pst* infection, indicating augmented activation of SA-mediated defense signaling. Interestingly, *TaPsIPK1* knockout plants exhibited comparable PTI defense gene expression and MAPK activation as WT plants in the absence of chitin treatment, while treatment of *TaPsIPK1*KO with chitin also resulted in accelerated PTI defense gene expression and MAPK activation. These results suggest that knockout of *TaPsIPK1* leads to the priming of immune defenses, which is rapidly amplified upon infection. This may explain why the *TaPsIPK1* mutation resulted in a stronger defense response without fitness penalty.

Although *S* genes are known to be used by pathogen effectors for pathogen penetration, proliferation, nutrient uptake, or inhibition of host immune response, the pathogen effectors manipulating *S* genes remain largely unknown. For example, *Mlo* (*Mildew resistance locus O*) is a well-known *S* gene that controls resistance to powdery mildew in barley and wheat (Wolter et al., 1993; Li et al., 2022), but pathogen effectors that manipulate its function have not yet been identified. Using a yeast two-hybrid (Y2H) assay, Wang et al. (2022) identified PsSpg1, an effector protein that is secreted by Pst and targets TaPsIPK1. TaPsIPK is located at the plasma membrane (PM) and has auto-phosphorylation activity. The interaction with PsSpg1 increases the phosphorylation activity of TaPsIPK1 and promotes the translocation of TaPsIPK1 from the PM into the nucleus. Intriguingly, translocation to the nucleus is essential for TaPsIPK1 to function as a susceptibility factor. As an important effector for *Pst* virulence, PsSpg1 requires the presence of TaPsIPK1 to promote *Pst* pathogenicity. TaPsIPK1 also interacts with Spg1 proteins from *P. triticina* but not from *P. graminis*, which correlated with the reduced susceptibility of the *TaPsIPK1*KO mutant to *P. triticina* but not to *P. graminis*. These data showed that *TaPsIPK1* is an important *S* gene specifically targeted by the Spg1 of *Pst* and *P. triticina*. *TaPsIPK1* is the first wheat *S* gene known to be hijacked by rust fungal effectors.

Having found that wheat susceptibility to *Pst* depends on the movement of TaPsIPK1 into the nucleus, Wang et al. (2022) then investigated the function of TaPsIPK1 in the nucleus of wheat cells. In Y2H screens, two wheat nuclear wheat proteins were also identified, including one transcription factor, i.e., TaCBF1d, which was further verified to interact with TaPsIPK1. In vitro biochemical assays showed that TaPsIPK1 phosphorylates TaCBF1d, and that the presence of PsSpg1 enhances the phosphorylation of TaCBF1d. Furthermore, the authors found that the phosphorylation level of TaCBF1d affects its role in transcriptional regulation. Increased phosphorylation of TaCBF1d results in decreased transcription of defense-related genes and increased transcription of the *S* gene *TaPsIPK1*, thereby increasing wheat susceptibility to the rust disease. These results illustrate the phosphorylation and transcriptional regulatory role of PsSpg1-TaPsIPK1-TaCBF1d in mediating wheat susceptibility to the rust fungi.

In summary, Wang et al. (2022) provide the first report of an *S* gene in wheat that can be used for resistance improvement. To their credit, the authors carried out extensive mechanistic studies on wheat susceptibility to rust despite the lack of genetic transformation of *Pst* and despite the complexity of the wheat genome. By inactivating the *S* gene *TaPsIPK1*, the authors establish an effective strategy for enhancing broad-spectrum resistance against *Pst* and *Ptt* with no adverse effects on wheat growth and yield. The results of this study suggest that high yielding wheat varieties that have lost their resistance because of pathogen adaptation can be improved through the modification of *S* genes. The study by Wang et al. (2022) therefore represents a major advancement in understanding plant–pathogen interactions and in applying that understanding for breeding disease-resistant crops.

## Data Availability

Not applicable.

## Abbreviations

*Pst*: *Puccinia striiformis* f. sp. *Tritici*
*Yr*: Wheat yellow rust resistance genes
*TaPsIPK1*: *Puccinia striiformis-Induced Protein Kinase 1*
CRISPR/Cas9: Clustered regularly interspaced short palindromic repeats/CRISPR-associated 9
PTI: PAMP-triggered immunity
ETI: Effector-triggered immunity
*Mlo*: *Mildew resistance locus O*
SA: Salicylic acid
Y2H: Yeast two-hybrid
